# Connexins and pannexins in liver damage

**DOI:** 10.17179/excli2016-119

**Published:** 2016-02-25

**Authors:** Sara Crespo Yanguas, Joost Willebrords, Michaël Maes, Tereza Cristina da Silva, Isabel Veloso Alves Pereira, Bruno Cogliati, Maria Lucia Zaidan Dagli, Mathieu Vinken

**Affiliations:** 1 Department of In Vitro Toxicology and Dermato-Cosmetology, Faculty of Medicine and Pharmacy, Vrije Universiteit Brussel, Laarbeeklaan 103, 1090 Brussels, Belgium; 2 Department of Pathology, School of Veterinary Medicine and Animal Science, University of São Paulo, Av. Prof. Dr. Orlando Marques de Paiva 87, São Paulo SP CEP 05508-900, Brazil

**Keywords:** connexin, pannexin, acute liver injury, steatosis, hepatitis, cholestasis, fibrosis, liver cancer

## Abstract

Connexins and pannexins are key players in the control of cellular communication and thus in the maintenance of tissue homeostasis. Inherent to this function these proteins are frequently involved in pathological processes. The present paper reviews the role of connexins and pannexins in liver toxicity and disease. As they act both as sensors and effectors in these deleterious events connexins and pannexins could represent a set of novel clinical diagnostic biomarkers and drug targets.

## Abbreviations

ATP: adenosine triphosphate, Cx: connexin, GJIC: gap junctional intercellular communication, HCC: hepatocellular carcinoma, NAFLD: non-alcoholic fatty liver disease, NASH: non-alcoholic steatohepatitis, Panx: pannexin

## Introduction

**Figure 1:**
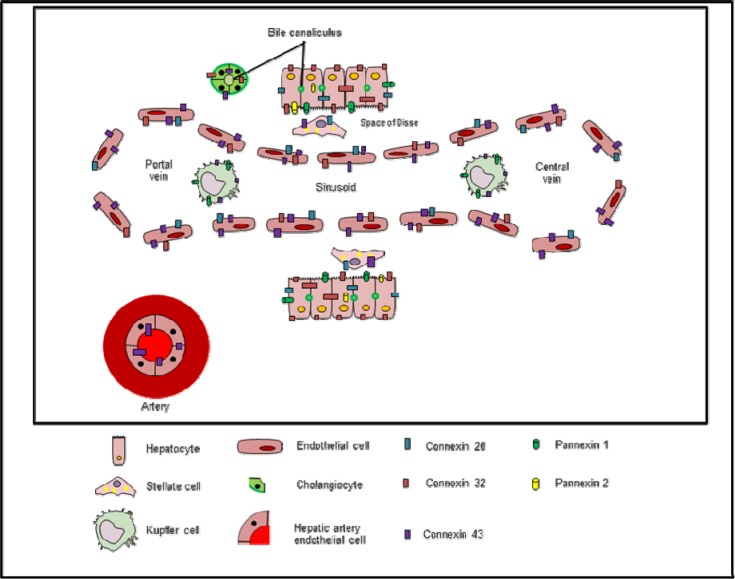
Expression of connexins and pannexins in liver cells

Like in other organs liver homeostasis relies on the interplay between extracellular, intracellular and intercellular signaling. The latter is mediated by gap junctions which arise from the interaction of 2 hemichannels, also called connexons of adjacent cells, each connexon being composed of 6 connexin (Cx) proteins. More than 20 different connexin variants have been identified, all which are named after their molecular weight as predicted by cDNA sequencing and expressed in kilodaltons (Bai and Wang, 2014[4]), and that are produced in a tissue-specific way. In liver, 5 different connexin species are detectable (Figure 1(Fig. 1)). Parenchymal liver cells, the hepatocytes, abundantly produce Cx32 and small quantities of Cx26. By contrast, Cx43 is the predominant connexin species present in nonparenchymal liver cells, including stellate cells, Kupffer cells, and sinusoidal endothelial cells (Fischer et al., 2005[22]). Cx37 and Cx40 are the major connexins harbored by liver vasculature (Chaytor et al., 2001[10]; Fischer et al., 2005[22]; Hernández-Guerra et al., 2014[29]; Shiojiri et al., 2006[67]). Nevertheless, gap junctions are mainly, if not uniquely, found between hepatocytes (Spray et al., 1994[70]). Gap junctions provide a pathway for the intercellular flux of small and hydrophilic substances, including adenosine triphosphate (ATP), cyclic adenosine monophosphate and inositol triphosphate, as well as several ions (Alexander and Goldberg, 2003[2]; Dbouk et al., 2009[16]; Decrock et al., 2009[17]). By doing so, gap junctional intercellular communication (GJIC) has been found critical for the performance of vital functions in liver, such as plasma protein synthesis (Yang et al., 2003[87]) and xenobiotic biotransformation (Neveu et al., 1994[50]; Shoda et al., 1999[68], 2000[69]).

About 15 years ago, a novel group of connexin-like proteins was discovered, the pannexin (Panx) family, with 3 members characterized thus far. Pannexins do not form gap junctions, but rather assemble in a configuration reminiscent of connexin hemichannels. They facilitate paracrine signaling by controlling the exchange of substances like ATP between the cytosol and the extracellular environment (Panchin et al., 2000[56]). A number of reports published in recent years have demonstrated Panx1 expression in liver tissue, in particular produced by hepatocytes (Bruzzone et al., 2003[8]; Csak et al., 2011[14]; Ganz et al., 2011[26]; Kim et al., 2015[34]; Xiao et al., 2012[83]) and Kupffer cells (Sáez et al., 2014[64]). Other studies showed the presence of Panx2 protein in mouse liver (Le Vasseur et al., 2014[40]) and rat hepatocytes (Li et al., 2008[43]) (Table 1(Tab. 1)) (References in Table 1: Hepatocyte: Bruzzone et al., 2003[8]; Csak et al., 2011[14]; Fischer et al., 2005[22]; Fowler et al., 2013[23]; Ganz et al., 2011[26]; Kim et al., 2015[34]; Kumar and Gilula, 1986[38]; Kuraoka et al., 1993[39]; Le Vasseur et al., 2014[40]; Li et al., 2008[43]; Nicholson et al., 1987[51]; Paul, 1986[58]; Xiao et al., 2012[83]; Zhang and Nicholson, 1989[90]; Kupffer cell: Eugenin et al., 2007[20]; Fischer et al., 2005[22]; Sáez et al., 2014[64]; Stellate cell: Fischer et al., 2005[22]; Hernández-Guerra et al., 2014[29]; Sinusoidal endothelial cell: Fischer et al., 2005[22]; Hernández-Guerra et al., 2014[29]; Cholangiocyte: Bode et al., 2002[6]; Hepatic artery endothelial cell: Chaytor et al., 2001[10]; Hernández-Guerra et al., 2014[29]; Shiojiri et al., 2006[67]). Although their physiological roles in liver remain to be established, pannexin-mediated communication has already been associated with liver pathology. In fact this paper will review the current knowledge regarding the involvement of connexins, pannexins and their channels in liver injury, *in casu* occurring in the context of liver disease and toxicity.

## Acute Liver Injury

Acute liver failure is a clinical syndrome from a variety of causes resulting from rapid loss in hepatocyte function, typically associated with coagulopathy and encephalopathy in a patient without preexisting liver disease. Upon administration of prototypical liver toxicants including thioacetamide, acetaminophen, *D*-galactosamine or carbon tetrachloride, to Cx32-lacking rodents, decreased aminotransferase serum levels and less liver damage is observed in comparison with wild-type littermates (Asamoto et al., 2004[3]; Naiki-Ito et al., 2010[47]; Patel et al., 2012[57]). Along the same line, hepatocytes originating from Cx32-deficient mice show reduced cell death when treated with acetaminophen *in vitro* (Saito et al., 2014[65]). This points to a role for Cx32-based signaling either in spreading noxious messengers or in the removal of dead cells in order to restore the homeostatic balance. In contrast to this is a recent report, describing protective effects of Cx32 in acetaminophen-triggered liver toxicity, possibly linked to the trafficking of glutathione between hepatocytes *via* gap junctions (Igarashi et al., 2014[30]). This can be reconciled with the well-known decay of Cx32 production and concomitant reduced channel activity upon exposure of hepatocytes to liver toxicants both *in vitro* and *in vivo* (Vinken et al., 2009[77]; Maes et al., 2016[45]). Hepatocellular gap junctions persist in the early phases of centrilobular necrotic cell death induced by thioacetamide in rat, yet they fade away during the subsequent restorative proliferative response. In a later stage, gap junctions initially emerge in perinecrotic areas and ultimately in all zones (Kojima et al., 1994[35]). Of note, in liver of rodents overdosed with acetaminophen, Cx43 is upregulated and *de novo* expressed in hepatocytes (Naiki-Ito et al., 2010[47], Maes et al., 2016[45]). In rat liver, this Cx43 expression is colocalized with caspase 3, suggesting a role for Cx43 in cell death. However, a recent study showed that Cx43-deficient mice display increased liver cell death, inflammation and oxidative stress in comparison to wild-type littermates after acetaminophen overdose (Maes et al., 2016[45]). Furthermore, high Cx43 immunoreactivity is observed around inflamed and necrotic areas in a rat model of acute-on-chronic liver failure (Balasubramaniyan et al., 2013[5]).

## Liver Steatosis

Non-alcoholic fatty liver disease (NAFLD) is currently the most common chronic liver disease worldwide. NAFLD is defined by the presence of liver fat accumulation exceeding 5 % of hepatocytes in the absence of significant alcohol intake. As such, NAFLD encompasses a broad histopathological spectrum, ranging from steatosis to non-alcoholic steatohepatitis (NASH) and even liver cancer (Loomba and Sanyal, 2013[44]; Willebrords et al., 2015[81]). NASH relies, at least in part, on the activation of inflammasomes, being multiprotein complexes involved in innate immunity and caspase 1 processing, which in turn leads to cleavage and extracellular release of interleukin 1 beta and interleukin 18 (Wree et al., 2014[82]). Panx1 channels have been repeatedly found to facilitate inflammasome activation (Pelegrin and Surprenant, 2006[61], 2007[60]). As a matter of fact, these pores support ATP release during lipoapoptosis induced by saturated free fatty acids in cultured hepatocytes, which is a hallmark of NASH. Panx1 channels are therefore thought to play a critical role in inflammation associated with lipotoxic liver injury (Xiao et al., 2012[83]). Interestingly, administration of lipopolysaccharide (Ganz et al., 2011[26]) as well as ischemia-reperfusion (Kim et al., 2015[34]) elevate hepatic Panx1 levels in mice.

## Hepatitis

Hepatitis refers to a general inflammatory response of the liver to a number of factors, such as drugs or viruses (Vinken et al., 2013[78]). Hepatitis patients present reduced amounts of Cx32 in the liver (Nakashima et al., 2004[48]; Yamaoka et al., 2000[85]), a feature that can be reproduced in rodents when treated with lipopolysaccharide (Correa et al., 2004[12]; Gonzalez et al., 2002[27]; Temme et al., 2000[71]). Deterioration of Cx32 expression hereby results from mRNA degradation (Theodorakis and De Maio, 1999[73]). Downregulation of Cx32 production by proinflammatory cytokines in cultures of primary hepatocytes is controlled by nuclear factor kappa beta signaling and mitogen-activated protein kinase, and is accompanied by abrogation of GJIC (Yamamoto et al., 2004[84]). Hepatic Cx26, however is positively affected by proinflammatory stimuli both *in vitro* and *in vivo* (Temme et al., 2000[71], 1998[72]). Likewise, Cx43 expression and GJIC become enhanced in cultures of primary stellate cells and Kupffer cells in inflammatory conditions (Eugenin et al., 2007[20]; Fischer et al., 2005[22]). Cx43 hereby moves from the cytosol to the membrane surface in order to assemble into functional gap junctions. Upregulated Cx43 production also occurs during liver inflammation *in vivo* (Eugenin et al., 2007[20]; Gonzalez et al., 2002[27]). This is thought to reflect the activation of Kupffer cells, which assists in the removal of debris and apoptosis of damaged hepatocytes following inflammation (Eugenin et al., 2007[20]). 

## Cholestasis

Acute or chronic impairment of bile flow from the liver to the duodenum is referred to as cholestasis. Upon cholestasis, hepatocytes adopt a brownish-green stippled appearance within the cytoplasm, which reflects bile accumulation. Canalicular bile plugs between hepatocytes or within bile ducts may also be observed, representing bile that has been excreted and that is obstructed in the duct. Because of increased pressure, such bile duct plugs may cause rupture and hence spilling of bile into surrounding tissue. This can induce hepatic necrosis and inflammation (Vinken et al., 2013[78]). Cholestasis can be experimentally induced by bile duct ligation. This is associated with decreased gap junction quantities and low Cx32 amounts in the liver (Balasubramaniyan et al., 2013[5]; Fallon et al., 1995[21]; Gonzalez et al., 2002[27]; Kojima et al., 2003[36]), which is mediated by the p38 mitogen-activated protein kinase (Kojima et al., 2003[36]). Cx26 levels also drop, while Cx43 production increases following bile duct ligation (Balasubramaniyan et al., 2013[5]; Fallon et al., 1995[21]). 

## Liver Fibrosis

Fibrosis is a wound-healing response to various types of injury, whereby quiescent stellate cells transform into proliferative, fibrogenic and contractile myofibroblast-like cells. This is associated with a cascade of biochemical events, such as proinflammatory cytokine release and extracellular matrix deposition, all which result in drastic phenotypic changes, including scarring. The final stage of fibrosis is called cirrhosis and is considered irreversible (Crespo Yanguas et al., 2016[13]; Friedman, 2008[25], 2010[24]; Lee et al., 2015[41]). Cx32 steady-state protein levels are reduced in cirrhosis patients, a process that goes hand in hand with its relocalization in the cytoplasm of hepatocytes (Nakashima et al., 2004[48]; Yamaoka et al., 2000[85], 1995[86]). Furthermore, upregulated Cx43 production has been observed in human cirrhotic liver tissue (Hernández-Guerra et al., 2014[29]). These findings are identical to those in rodents following chronic administration of thioacetamide or carbon tetrachloride (Nakata et al., 1996[49]). In Cx43-lacking mice, strongly reduced cell death and hepatocellular injury is observed after treatment with carbon tetrachloride (Cogliati et al., 2011[11]). The latter induces translocation of both Cx26 and Cx43 from the plasma membrane to the cytoplasm and nuclei of sinusoidal endothelial cells, a scenario that is equally seen for Cx32 in hepatocytes. Perinuclear residing of Cx26 and Cx43 also occurs in cultures of spontaneously activated primary stellate cells (Fischer et al., 2005[22]). This could underlie the establishment of heterologous communication between stellate cells and hepatocytes under these conditions (Rojkind et al., 1995[62]), whilst homologous GJIC in cultured hepatocytes is suppressed by carbon tetrachloride (Saez et al., 1987[63]). Collectively, these observations suggest distinct roles for connexins in each liver cell type in the process of fibrogenesis (Oloris et al., 2007[53]).

## Liver Cancer

Chronic liver disease may burgeon into the onset of liver cancer, mainly hepatocellular carcinoma (HCC). GJIC is strongly reduced in HCC cells (Mesnil et al., 2005[46]; Yang et al., 2003[87]; Yano et al., 2001[88]). This is paralleled by cytoplasmic Cx32 localization, which is believed to promote motility and metastatic potential (Li et al., 2007[42]). Decrease of Cx26 production in HCC has been related to epigenetic modifications, in particular DNA methylation (Shimizu et al., 2007[66]; Tsujiuchi et al., 2007[74]). Concomitantly, Cx43 gradually appears in the cytoplasm and at the plasma membrane of HCC cells (Krutovskikh et al., 1994[37]; Oyamada et al., 1990[55]; Wang et al., 2013[80]). In fact, the extent of cytoplasmic Cx43 localization corresponds with the malignant potential of the liver tumor (Kawasaki et al., 2007[33]). In addition, Cx43 expression in HCC is linked to migration, invasion and metastatic ability (Ogawa et al., 2012[52]). Silencing of Cx43 production in liver cancer cells inhibits proliferation and favors the differentiated phenotype, whereas the opposite has been observed in HCC cells that artificially overexpress Cx43. Not surprisingly, Cx32 amounts and gap junction activity inversely correlate with Cx43 presence in HCC cells. Cx43 is therefore considered a hepatic oncogene (Zhang et al., 2007[89]). By contrast, Cx32 acts as a liver tumor suppressor, a notion that is supported by the observation that Cx32 knockout rodents display increased susceptibility to chemically induced hepatocarcinogenesis (Dagli et al., 2004[15]; Igarashi et al., 2013[31]).

## Conclusions

Because of its unique localization and position in the organism, the liver is a major target for systemic toxicity and disease (Vinken et al., 2013[78]). Connexins are goalkeepers in hepatic homeostasis and hence are routinely involved in liver pathology. They act both as sensors and effectors in this process. Regarding the former, a general observation in liver disease is that Cx32 production gradually decreases at the expense of Cx43 (Krutovskikh et al., 1994[37]; Oyamada et al., 1990[55]; Wang et al., 2013[80]). This renders Cx43 a potential biomarker that can be used for diagnostic purposes. In addition, connexins can also represent drugable targets due to their active role in liver pathogenesis. Research in this direction is nowadays challenged with the complex multifaceted communication capacities of connexins. Indeed, in the last decade, it has become clear that connexin hemichannels not only are the structural building blocks of gap junctions, but also are equally signaling entities on their own. They specifically establish a circuit for trafficking of messengers, such as ATP, between the cytosol and the extracellular space, similar to pannexin-based communication. Unlike their full channel counterparts however, hemichannels have a low open probability (Chandrasekhar and Bera, 2012[9]; D'hondt et al., 2014[18]; Decrock et al., 2009[17]). In fact, although heavily debated and still highly criticized, it seems that hemichannels specifically open during pathological circumstances, which is another difference with gap junctions. In this respect, hemichannels consisting of Cx32 and to a lesser extent of Cx43, but not their corresponding gap junctions have been found to drive hepatocyte cell death (Vinken et al., 2010[75], 2012[76]). Therefore, inhibition of hemichannels could introduce a novel strategy for the clinical management of liver disease. This also holds true for pannexin channels that underlie inflammatory processes, including in liver disease (Csak et al., 2011[14]; Diezmos et al., 2013[19]; Ganz et al., 2011[26]; Gulbransen et al., 2012[28]; Xiao et al., 2012[83]). Focus should thereby be put on the development of pharmacological inhibitors of hemichannels and pannexin channels. Most of the currently available inhibitors of these channels are not able to distinguish between connexin and pannexin signaling on the one hand and between hemichannel communication and GJIC on the other hand (Bodendiek and Raman, 2010[7]). An exception includes the group of so-called mimetic peptides, which reproduce specific amino acid sequences in the connexin protein structure. Some of these mimetic peptides have the ability to inhibit hemichannels without affecting gap junctions (Abudara et al., 2014[1]; Iyyathurai et al., 2013[32]) and have been found to protect against cell death *in vivo* (Wang et al., 2013[79]). Similarly, specific pannexin mimetic peptides are able to counteract inflammation and cell death (Orellana et al., 2011[54]; Pelegrin et al., 2008[59]). Such compounds should be further explored in future, as they may open new avenues for the clinical treatment of liver disease.

## Notes

Sara Crespo Yanguas and Joost Willebrords are equally contributing first authors.

Maria Lucia Zaidan Dagli and Mathieu Vinken share equal seniorship.

## Acknowledgements

This work was financially supported by the grants of Agency for Innovation by Science and Technology in Flanders-Belgium (IWT) the University Hospital of the Vrije Universiteit Brussel-Belgium (“Willy Gepts Fonds” UZ-VUB) the Fund for Scientific Research Flanders-Belgium (FWO grants G009514N and G010214N) the European Research Council (ERC Starting Grant 335476) the University of São Paulo-Brazil and the Foundation for Research Support of the State of São Paulo-Brazil (FAPESP SPEC grant 2013/50420-6).

## Conflict of interest

The authors declare that they have no conflict of interest.

## Figures and Tables

**Table 1:**
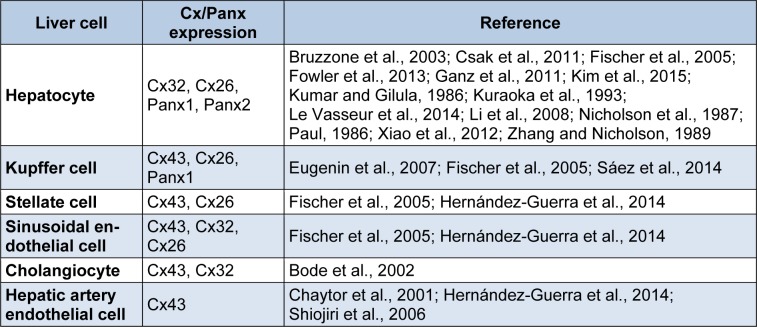
Expression of connexins and pannexins in liver cells
